# Metagenomic Survey of Tomato Rhizosphere Microbiome Using the Shotgun Approach

**DOI:** 10.1128/mra.01131-21

**Published:** 2022-02-03

**Authors:** Olubukola Oluranti Babalola, Afeez Adesina Adedayo, Ayomide Emmanuel Fadiji

**Affiliations:** a Food Security and Safety Niche, Faculty of Natural and Agricultural Sciences, North-West University, Mmabatho, South Africa; Queens College CUNY

## Abstract

Food sustainability, e.g., fruit and vegetables, is a major agricultural problem that requires monitoring. Rhizosphere microbiomes’ abundance and functionality are essential in promoting tomato plants’ growth and health. We selected farms in South Africa’s North West Province and present the metagenomes of their tomato rhizospheres and associated functional potentials.

## ANNOUNCEMENT

The North West Province is a semiarid region with high temperatures. The soil in this region is populated by important microorganisms, with essential characteristics that promote the planting of tomatoes (1). Tomatoes produce annual crops of about 600,000 tonnes and find their way to South Africa through Europe and South American countries like Peru and Ecuador (2). The availability of these fruits in South Africa will enhance food production for human consumption because of their richness in essential vitamins and carotenoids (3). Three main cultivars of tomatoes are predominant in South Africa, namely, round or fresh tomatoes, Roma tomatoes, and cherry tomatoes, contributing about 24% of the total vegetable production in South Africa (4). Tomatoes are cultivated in open fields under irrigation in the following South African provinces: Eastern Cape, Western Cape, northern Kwazulu-Natal, North West, Mpumalanga, and Limpopo.

We collected the soil samples used in this study from the root region of Roma tomato plants at the experimental farm of the North-West University, Mafikeng (26°019′36.9″S, 26°053′19.0″E; 25°47′19.1″S, 25°37′05.1″E; 25°47′17.0″S, 25°37′03.2″E; altitude, 159 km). The site has a 450-mm annual rainfall record and regional temperatures ranging from 25°C to 37°C (5). The experimental soil samples were collected from the rhizosphere of healthy and diseased tomato plants and the bulk soil. Bulk soil, which served as the control soil, was collected from a natural grassland with no tomato plantation, 20 m from the tomato plantation field. We took rhizosphere soil from the Roma tomato plants from three different areas of a site. Fifteen samples were put in separate sterile polyethene bags, kept in a cold box at −4°C, and transported to the laboratory. All collected soil samples were then stored at a temperature of −20°C before extraction of DNA for shotgun metagenomic sequencing.

From the stored rhizosphere soil, 5 g from each sample was measured using a calibrated scale. DNA was extracted using the NucleoSpin Soil kit (Macherey-Nagel, Germany). The quality of the extracted DNA was assessed using a NanoDrop spectrophotometer.

The libraries were prepared with 50 ng DNA using a Nextra DNA Flex kit, undergoing fragmentation and the ligation of adapter sequences. The final concentrations of the libraries were measured using the Qubit double-stranded DNA (dsDNA) HS assay kit (Life Technologies), and the mean lengths of the DNA fragments were ascertained using a 2100 Bioanalyzer (Agilent Technologies). The libraries were then monitored, combined at 0.6 nM, and sequenced using a NovaSeq 6000 system (Illumina) with 300 cycles.

SolexaQA v1.6 was used to conduct the quality control (QC) of raw data, reduce low-quality reads, and remove replicate data (6). Duplicate read inferred sequencing error estimation (DRISEE) enables us to assess the error of sequenced samples caused by artificial replicated sequenced data (7). We employed the default settings of the MG-RAST v4.0.3 server to perform analytical processing downstream (8, 9) (Table 1).

**TABLE 1:** Sequence reads for the rhizosphere soil samples analyzed

Sample^*a*^	Data before QC					Data after processing		Data after QC				Data after alignment	
	Size (bp)	No. of sequence reads	Mean GC content (%)	Mean sequence length (bp)	No. of artificial duplicate read sequences	No. of known proteins predicted	No of RNA features predicted	Size (bp)	No. of sequence reads	Mean GC content (%)	Mean sequence length (bp)	No. of known proteins identified	No of RNA features identified
HT21 (HR)	2,152,004,650.3	13,739,258.3	64 ± 10	154 ± 32	836,435	7,850,484.3	28,853	765,041,235.3	12,665,143.7	63 ± 9	155 ± 33	3,985,525.7	5,962.7
HT21 (DR)	1,409,528,303.3	19,765,082	64 ± 10	155 ± 32	757,641.7	4,208,873.7	30,027	1,352,124,415	11,966,279.7	64 ± 9	156 ± 39	4,178,206.3	7,656.7
HT21 (BR)	1,477,197,003	138,145,859.3	65 ± 9	155 ± 32	374,748	11,448,097.3	25,322.7	1,385,218,693	5,247,459	64 ± 9	156 ± 34	2,515,439.7	5,439.3

a HR, healthy rhizosphere; DR, diseased rhizosphere; BR, bulk rhizosphere.

**FIG 1:**
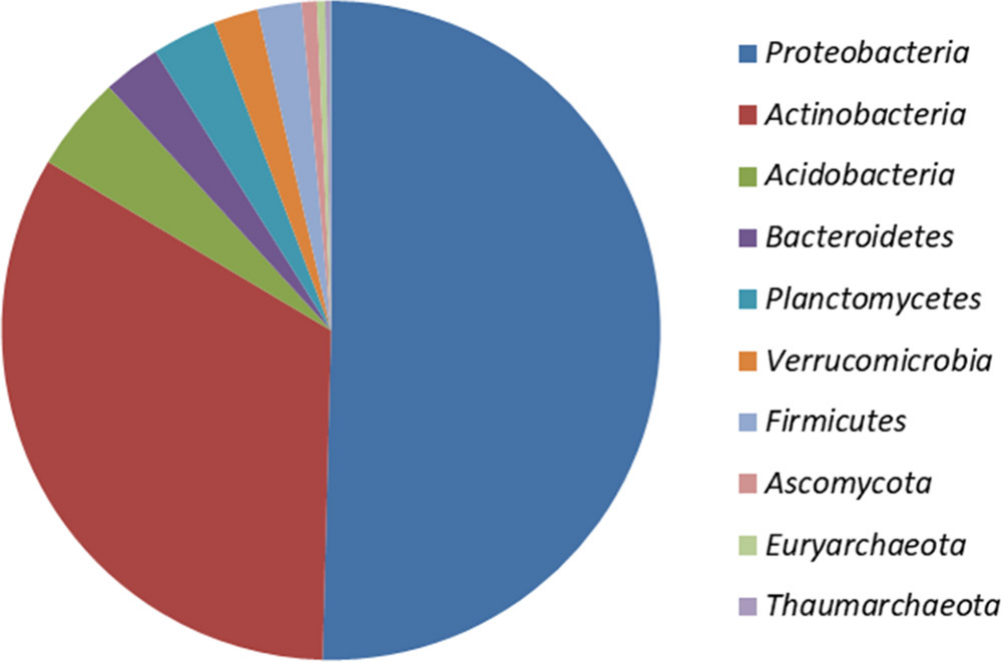
Abundant phyla obtained according to the taxonomic system.

The domain or kingdoms obtained according to the taxonomic system are *Bacteria*, *Eukaryota*, and *Archaea*. The most abundant phyla belonged to the *Bacteria* domain; *Proteobacteria* (38.8 to 54%) and *Actinobacteria* (25.4 to 35.5%) were the most abundant, and others, such as *Acidobacteria* (2.3 to 5.2%), *Bacteroidetes* (3.0 to 3.9%), *Planctomycetes* (2.6 to 3.4%), *Verrucomicrobia* (2.2 to 2.3%), and *Firmicutes* (1.7 to 2.4%), were also significant. Moreover, reads for fungi (*Ascomycota* and *Basidiomycota*) and archaea (*Thaumarchaeota* and *Euryarchaeota*) were also identified but at <1% relative abundance (Fig. 1).

Functional annotation after mapping with SEED subsystems (10) revealed the presence of the following important attributes: carbohydrates (13.2 to 14.8%), clustering-based systems (12.7 to 12.8%), amino acids and derivatives (10.1 to 10.3%), protein metabolism (8.2 to 8.3%), DNA metabolism (4.3 to 4.6%), cell wall and capsule (3.4 to 3.6%), RNA metabolism (3.3 to 3.5%), and stress response (2.5 to 2.7%).

### Data availability.

The metagenomes of rhizosphere soil sequence reads were submitted to the NCBI with BioProject accession number PRJNA766489 and Sequence Read Archive (SRA) accession numbers SRX12366062, SRX12366063, and SRX12366064 (healthy), SRX12366065, SRX12366066, and SRX12366067 (diseased), and SRX12366068, SRX12366069, and SRX12366070 (bulk).
